# A Point in the Treatment of Hip-joint Disease

**Published:** 1881-04

**Authors:** Norman H. Chapman

**Affiliations:** Late House Surgeon to the Philadelphia Orthopædic Hospital, etc.; Philadelphia


### ARTICLE V.

A POINT IN THE TREATMENT OF HIP JOINT DISEASE. By NORMAN H. CHAPMAN, M. D., M. S., late House Surgeon to the Philadelphia Orthopædic Hospital, etc.

The old plan of treating coxalgia by long continued rest in bed with weight extension, while it is correct in principle, is at the same time a great tax upon the general health of a patient. Persons of a naturally strong constitution may, under proper conditions, be put to bed for a considerable period and not overtax their powers of endurance, but with the average victims of hip joint disease it is highly prejudicial to confine them to bed for a longer time than is absolutely necessary to get control of the acute inflammatory symptoms. The condition of the general health bears such an intimate relation to the onward progress of the disease that it should receive every possible attention.

During the first stage of this affection when the joint inflammation is acute, there is unquestionably no better mode of procedure than by giving the parts rest in bed, separating the articulating surfaces by weight extension, and the local application of croton oil, the hot iron, or some other powerful counter-irritant. This plan, however, must not be persisted in too long. In fact just as soon as the acute inflammatory symptoms have been placed under control, it is good surgery to get the case up and about, keeping it as much as possible in the open air. Out-of-door exercise and the liberal use of such a tonic and dialectic regimen as will best tend to build up and invigorate the general health should be encouraged. No particular which tends to elevate its general tone is beneath consideration.

The value of early getting a case of hip joint disease out of bed and into the open air was very clearly set forward a few years ago by the advocates of the treatment of such cases by means of a high heel and sole upon the well side, together with a heavy mechanical appliance attached to the affected limb. The principle involved in this plan of treatment—*i. e.*, that of swinging the limb—is a good one, and the point to which I desire, at this time, to call your attention is the ready means by which it can be carried out with the aid of a fixed dressing and a pair of crutches.

The mode of applying the fixed dressing referred to is simple. The leg of the affected side is flexed upon its thigh to a little more than a right angle, and is then encased in either a silicate of soda, plaster of paris, or glue and acetate of lead permanent dressing. After the dressing has been allowed to harden it is cut open on one side, and should be provided with shoe-hooks, or eyelet holes and laces, so that it can be removed at will. During the day the patient wears the fixed dressing and walks about upon crutches. The foot of the affected side does not touch the ground, the limb swings, and the combined weight of the lower extremity and dressing keep up constantly a sufficient amount of extension to prevent the articulating surfaces of the joint from rubbing against each other. During the night, when the patient is in the recumbent posture and the influence of gravity is removed so that no extension is produced,the fixed dressing should be removed and a weight extension apparatus applied. For this purpose the Morton extension apparatus* is very convenient, as it is quickly taken off and re-applied. Weight extension by means of the adhesive strips answers every purpose, however, when the Morton apparatus is not at hand. The ankle knot may also be used, and is frequently all that is required for the over-night extension.

There have been a variety of braces and mechanical appliances brought to the notice of the medical profession for the treatment of coxalgia, and each from time to time have had their especial advocates. While I do not in the least deprecate the use of such apparatus—and especially good is the one made by Messrs. D. W. Kolbé & Son, of this city,—still they are all (that is those which are of any use) far too expensive to be employed by the general practitioner in the lower walks of life. The call is for something which does the work of a costly extension apparatus, and is at the same time within the reach of all. The fixed dressing which I have described would seem to fulfill this indication. It is simple,

* Surgery at the Pennsylvania Hospital, P.

comparatively inexpensive, and from abundant hospital experiment has been found to be equally efficient with the most approved methods of treatment by mechanical apparatus now before the profession.

Philadelphia, March, 1881.

